# Differences in gram-positive bacterial colonization and antimicrobial resistance among children in a high income inequality setting

**DOI:** 10.1186/s12879-019-4104-2

**Published:** 2019-05-29

**Authors:** Felipe Piedade Gonçalves Neves, Mariel Asbury Marlow, Gabriel Rezende-Pereira, Marcos Gabriel Pinheiro, Allyne Fandino Martinez dos Santos, Maria de Fátima Nogueira de Freitas, Rosana Rocha Barros, Fábio Aguiar-Alves, Claudete Aparecida Araújo Cardoso, Lee Woodland Riley

**Affiliations:** 1 Instituto Biomédico, Universidade Federal Fluminense, Niterói, RJ Brazil; 2 School of Public Health, University of California, Berkeley, CA USA; 3 Laboratório Universitário Rodolpho Albino, Universidade Federal Fluminense, Niterói, RJ Brazil; 4 Faculdade de Medicina, Universidade Federal Fluminense, Niterói, RJ Brazil

**Keywords:** MRSA, Beta-hemolytic streptococci, Colonization, Health disparities, Income inequality

## Abstract

**Background:**

*Staphylococcus aureus* and beta-hemolytic streptococci (BHS) diseases disproportionately affect populations in middle/low-income countries. To assess if this disparity is reflected in colonization by these organisms, we compared their colonization frequency among children from different socioeconomic status (SES) communities in a city with high income inequality.

**Methods:**

Between May–August 2014, we collected nasal and throat swabs to investigate *S. aureus* and BHS colonization among children who attended private and public pediatric clinics. Patients were classified as high SES, middle/low SES, and slum residents. We investigated the antimicrobial resistance profile, the SCC*mec* types and the presence of PVL genes among methicillin-resistant *S. aureus* (MRSA). We also examined the antimicrobial resistance profile and serogroups of BHS.

**Results:**

Of 598 children, 221 (37%) were colonized with *S. aureus*, of which 49 (22%) were MRSA. MRSA colonization was higher in middle/low SES (*n* = 18; 14%) compared with high SES (*n* = 17; 6%) and slum (*n* = 14; 8%) residents (*p* = 0.01). All MRSA strains were susceptible to clindamycin, nitrofurantoin, and rifampin. The highest non-susceptibility frequency (42.9%) was observed to erythromycin. SCC*mec* type V was only found in isolates from high SES children; types I and II were found only in middle/low SES children. Ten (20%) MRSA isolates carried PVL genes. Twenty-four (4%) children were BHS carriers. All BHS (*n* = 8) found in high SES children and six (67%) isolates from slum patients belonged to group A. All group B streptococci were from middle/low SES children, corresponding to five (71%) of the seven BHS isolated in this group. BHS isolates were susceptible to all drugs tested.

**Conclusions:**

Children from different SES communities had distinct bacterial colonization profiles, including MRSA carriage. Public health officials/researchers should consider SES when assessing disease transmission and control measures.

## Background

*Staphylococcus aureus* and beta-hemolytic streptococci (BHS), including *Streptococcus pyogenes* (group A streptococci, GAS) and *Streptococcus agalactiae* (group B streptococci, GBS), are Gram-positive bacteria that cause a range of infections, from mild cutaneous and upper-respiratory infections to invasive bloodstream and respiratory infections [1–3]. They permeate our environment, and can be found in food, hospitals, schools, childcare centers, and households [4]. These bacteria, especially *S. aureus* and *S. pyogenes*, can also asymptomatically colonize the human upper respiratory tract and the skin, making their spread difficult to control in a population. In a susceptible host, colonization can be a major risk factor for disease [5].

In children, *S. aureus* is one of the organisms most frequently isolated from patients with healthcare-associated infections, causing not only skin and soft tissue infections, but also invasive infections, such as bacteremia, osteomyelitis and septic arthritis [6]. Despite the low mortality rate (1–2%) for hospitalized children with methicillin-resistant *S. aureus* (MRSA) infection, there seems to be an increasing trend in the incidence of MRSA infections in such children [7]. Also, MRSA bacteremia in pediatric patients has been frequently associated with treatment failure and complications [8].

Methicillin resistance is encoded by genes organized in the staphylococcal cassette chromosome *mec* (SCC*mec*). To date, 13 SCC*mec* types (I-XIII) have already been described [9]. In addition, community-associated MRSA (CA-MRSA) strains often contains the Panton-Valentine leucocidin (PVL), which may be associated with severe diseases, such as necrotizing pneumonia [10].

The prevalence of GAS infections is higher among children, which is considered as a ‘hazard’ in school-aged children [11]. In addition, 319,000 infants were estimated to have disease caused by GBS globally in 2015, and approximately 7000 of these patients presented with neonatal encephalopathy [12].

While these pathogens represent a major public health burden among pediatric populations globally, diseases caused by these pathogens disproportionately affect populations in middle- and low-income countries [13]. Resource-poor communities, such as urban slums, have conditions that enhance the spread of bacteria, including crowding, untreated water, open sewage, and poor access to or uptake of health care services [14]. Although now classified by the World Bank as an upper-middle-income country in 2014, Brazilian cities have very high income disparity, with slum and high-income communities located in close proximity. Despite a high gross domestic product (GDP) of $USD 1.79 trillion, over 11 million people live in informal settlements or slums in Brazil [15].

While the incidence of diseases caused by these Gram-positive bacteria differs between high-income vs. middle and low-income populations, colonization frequency and strain characteristics by socioeconomic status (SES) in children residing in the same city is not well characterized. As varying living conditions among communities may result in different disease prevalence and transmission pathways, we investigated *S. aureus* and BHS colonization patterns among children living in high and middle/low SES, and slum communities. We compared risk factors for colonization and drug resistance of the isolates by SES group in the same city.

## Methods

### Study sites and population

For this cross-sectional study, we recruited children who attended two private pediatric clinics and one public pediatric clinic for primary care or routine follow-up. Study clinics were located in the city of Niterói, in Rio de Janeiro metropolitan area, Rio de Janeiro state, Brazil. In 2014, the city had a population of approximately 488,000 [16] and a Gini index (an economic measure of inequality) of 0.60, a ranking consistent with Gini indexes of the 10 most unequal countries in the world [17]. The private clinics provide care for children who have private health insurance or who pay out-of-pocket; both clinics are located in the high-income neighborhood in the western section of the city. The public clinic provides free health care to any child through the Brazilian national public health care system (Unified Health System–*Sistema Único de Saúde* or SUS). The clinic is part of a primary care facility, located on the border between middle/low SES and slum neighborhoods in the eastern section of the city. The private clinics are 1.7 km apart from each other and approximately 8 km from the public clinic.

### Patient recruitment and specimen collection

All children less than 18 years of age were consecutively recruited from May 12th to August 12th, 2014. Each of the clinics were sampled alternately for three half-day recruitment sessions per week. The number of recruited children was monitored weekly to assure corresponding sample size recruitment in each clinic per week. Age-appropriate written assent was obtained for children 7 years of age or older and verbal assent for children under 7 years of age. Following legal guardians’ written consent, one nasal swab and one throat swab were obtained from each child. Swabs were immediately placed into a tube containing Stuart medium until transported to the laboratory on the same day. A questionnaire for clinical and socioeconomic data was completed by child’s legal guardian using the mobile data collection application Magpi (Magpi, Washington, DC).

### Laboratory processing and characterization of the isolates

We used the mannitol salt agar to isolate staphylococcal colonies from nasal swabs and we identified *S. aureus* using catalase and coagulase tests. Staphylococcal isolates were tested for antimicrobial resistance against 30-μg cefoxitin and 1-μg oxacillin by the disk-diffusion method to identify MRSA strains [18]. Resistance was then confirmed by the presence of the *mecA* gene via polymerase chain reaction (PCR) [19]. Multiplex PCR was used to assess SCC*mec* type for MRSA isolates [19]. Presence of PVL genes was investigated by PCR [20]. BHS colonies were detected by culture on 5% sheep blood agar plates and their phenotypic identification was performed by determining the group carbohydrate antigen (Lancefield groups A, B, C, F, and G), susceptibility to 0.04 U bacitracin and PYRase activity [21]. Antimicrobial susceptibility testing was performed by the disk-diffusion method on Mueller-Hinton agar (MHA) plate for MRSA strains and on MHA with 5% sheep blood agar plate for BHS [18]. The antimicrobial agents tested for MRSA were: 5-μg ciprofloxacin, 5-μg chloramphenicol, 2-μg clindamycin, 15-μg erythromycin, 10-μg gentamicin, 300-μg nitrofurantoin, 5-μg rifampin, 1.25/23.75-μg trimethoprim-sulfamethoxazole, and 30-μg tetracycline. For BHS, the following antimicrobial agents were tested: 30-μg ceftriaxone, 2-μg clindamycin, 15-μg erythromycin, 5-μg levofloxacin, 10-UI penicillin G, 30-μg tetracycline, and 30-μg vancomycin.

### Data analysis

We estimated that 600 children would be needed to detect a difference of 10% in MRSA and BHS colonization between private and public clinics, with a two-tailed α of 0.05 and a (1-β) of 0.80, for a comparison of two independent proportions. Final estimate accounts for a conservative 20% potential missing data/sample rate. This difference is based on previous studies on MRSA in the same city [22] and GAS in Salvador, Brazil [23].

For this analysis, we classified pediatric patients into three SES groups based on clinic attended and self-reported slum residence: high SES children were those who attended the private clinic and self-reported non-slum residence; middle/low SES were those children who attended the public clinic and self-reported non-slum residence; and slum children were those who attended either a public or private clinic and self-reported slum residence. To support the use of this SES classification, patient addresses were also geocoded with Google Maps API v3 and mapped in ArcGIS 10 (ESRI, Redlands, CA). Patient addresses were then overlaid with census tracts to determine proximity to or residence inside of a slum, defined by the Brazilian government as *aglomerados subnormais* (AGSN). An AGSN has at least 51 housing units on illegally occupied land with non-municipal approved construction or insecure access to essential public services [15].

Household salary is presented in USD based on the average USD:BRL exchange rate during the study period of 1:2.24 and categorized as multiples of the Brazilian monthly minimum wage in 2014 (USD $324).

We used Chi-squared tests, or Fisher’s exact tests (two-tailed) when appropriate, to assess associations between categorical variables and the outcomes of interest. We also used t-tests, or ANOVA tests when appropriate, to assess associations between continuous variables and the outcomes of interest. Variables found to have a statistically important association, defined as *p* < 0.10, were included in the regression analysis. Factors significant in the bivariate logistic regression or suspected to be potential confounding factors were included by step-wise variable selection to build the multivariate regression model. Lack of fit of the regression model was assessed by failure to reject the Hosmer and Lemeshow Goodness-of-Fit Test. Statistical analyses were carried out in SAS University Edition (SAS Institute Inc., Cary, NC).

## Results

Of 682 pediatric patients invited to participate, 598 (88%) consented and were enrolled, including 298 children from private clinics and 300 children from public clinic. Non-participation frequency was 10% (33/332) in the private clinics and 14% (50/350) in the public clinic. Most frequent reasons for non-participation included children who were vomiting or too fatigued to participate at recruitment, not enough time, and legal guardian under the age of 18 years.

Patient demographic characteristics were consistent with patient SES group, with high SES, middle/low SES, and slum groups, being significantly different across all demographic characteristics investigated except gender (Table 1). The mean age of children classified in high-income group was 3.2 years compared to 5.9 years for children from middle/low-income neighborhoods and 6.3 years for children from slum community (*p* < 0.01). Mean monthly income of slum residents was $669, compared to $1005 for middle/low-income and $3500 for high-income residents (*p* < 0.01). Slum residents were more likely to be geocoded as living in a slum census tract (AGSN) compared to other groups (*p* < 0.01).

**Table 1:** Patient characteristics by socioeconomic status for pediatric patients examined at private and public clinics – Niterói, RJ, Brazil, 2014

Demographic characteristic	High-income patient		Middle/low-income patient		Slum resident patient		Total		*P* value
	n	%	n	%	n	%	n	%	
Male	151	52	64	51	90	49	305	51	0.83
Mean age in years (SD)	3.2	4	5.9	5	6.3	5	4.8	5	< 0.01
Child race or ethnicity									< 0.01
White	209	72	52	42	58	32	319	53	
Mixed (*pardo* or *mestiço*)	63	22	58	46	108	59	229	38	
Black	18	6	15	12	17	9	50	8	
Attends childcare center	53	18	21	17	18	10	92	15	0.04
Child attends a public childcare center or school (vs private)	9	6	55	67	93	79	157	26	< 0.01
Stable income	270	93	105	85	130	71	505	84	< 0.01
Mean monthly household income in USD (SD)	3500	2985	1005	1277	669	500	2141	2578	< 0.01
Receives government financial assistance (*Bolsa Família*)	3	1	41	33	76	43	120	20	< 0.01
Geocoded distance to slum census tract									< 0.01
Within slum census tract	1	0.4	19	16	30	19	50	8	
Within 50 m	6	2	12	10	66	41	84	14	
Within 100 m	8	3	18	15	15	9	41	7	
More than 100 m	250	94	69	59	49	31	368	62	
Number of persons in household									< 0.01
2 (Single parent/guardian)	10	4	6	5	11	6	27	5	
3 or 4	225	78	74	59	109	60	408	68	
5 or more	55	19	45	36	63	34	163	27	
Other children 5 years or younger in household	55	19	37	30	55	30	147	25	0.01
Residence type									< 0.01
Apartment	100	35	2	2	0	0	102	17	
House	189	65	123	98	183	100	495	83	
Education of guardian									< 0.01
Illiterate or incomplete primary education	0	0	34	27	66	36	100	17	
Complete primary education or incomplete secondary education	4	1	35	28	44	24	83	14	
Complete secondary education or incomplete higher education	117	40	41	33	63	35	221	37	
Complete higher education	169	58	14	11	9	5	192	32	
Employment status of guardian									< 0.01
Employed	254	85	75	60	120	66	440	74	
Unemployed	8	3	9	7	8	4	25	4	
Stay-at-home parent/guardian	30	10	30	24	47	26	107	18	
Other (retired, student etc.)	7	2	11	9	6	3	24	4	
Animals living in the house	119	41	70	56	87	48	276	46	0.02
Total	290	49	125	21	183	31	598	100	

*SD* Standard deviation

Of the 598 children included in this study, 221 (37%) were colonized with *S. aureus*, of which 49 (22%) were colonized with MRSA. All MRSA strains were susceptible to clindamycin, nitrofurantoin, and rifampin. Lower resistance frequencies among MRSA were observed for ciprofloxacin, chloramphenicol (2% each), gentamicin, trimethoprim-sulfamethoxazole (6.1% each), and tetracycline (12.2%). Non-susceptibility to erythromycin made up 42.9%, comprising two (4.1%) intermediate and 19 (38.8%) resistant isolates. Multidrug resistance was observed in eight (16.3%) MRSA strains, which presented resistance to beta-lactams and to other two or three classes of antimicrobial agents. BHS colonization was observed in 24 (4%) of the 598 patients. All BHS isolates were susceptible to all antimicrobial agents tested.

### Differences in BHS serogroups and *S. aureus* SCC*mec* types

Table 2 shows the difference in colonization with BHS serogroups and MRSA SCC*mec* type distribution. BHS colonization did not differ significantly between SES (*p* = 0.30). However, BHS serogroup differed significantly among the three SES groups (*p* < 0.01). GAS was the most frequent BHS found among the population investigated, comprising 15 (62.5%) of the 24 isolates.

**Table 2:** Colonization with *Staphylococcus aureus* and beta-hemolytic streptococci and distribution of MRSA SCC*mec* types by socioeconomic status for pediatric patients examined at private and public clinics – Niterói, RJ, Brazil, 2014

Characteristic	High-income patient		Middle/low-income patient		Slum resident patient		*P* value
	n	%	N	%	N	%	
*S. aureus* colonization	94	32	51	41	76	42	0.09
MSSA colonization	77	27	33	26	62	34	0.19
MRSA colonization	17	6	18	14	14	8	0.01
MRSA SCC*mec* type							0.31
Type I	0	0	1	6	0	0	
Type II	0	0	1	6	0	0	
Type III	1	6	1	6	1	7	
Type IV	13	77	15	83	13	93	
Type V	3	18	0	0	0	0	
Presence of PVL gene in MRSA isolate	4	19	2	10	4	21	0.57
Streptococcal colonization	8	3	7	6	9	5	0.30
Streptococcal Lancefield group							0.001
Group A	8	100	1	14	6	67	
Group B	0	0	5	71	0	0	
Group C or G	0	0	1	14	3	33	

*MSSA* methicillin-susceptible *Staphylococcus aureus*, *MRSA* methicillin-resistant *Staphylococcus aureus*, *PVL* Panton-Valentine leucocidin

Colonization with *S. aureus* varied between 32 and 42% among SES groups, but it was not statistically significant (*p* = 0.09). However, colonization with MRSA was more common among middle/low-SES patients (*p* = 0.01). MRSA SCC*mec* type IV was the most frequent type across all three SES and all 10 (20.4%) isolates that had the PVL genes carried this type. All the SCC*mec* types found among the isolates, but type III, were associated with multidrug-resistant (MDR) strains. Resistant to gentamicin was only observed among the three MDR strains that carried the PVL genes.

### Risk factors for MRSA colonization

Income (*p* = 0.03), income stability (*p* = 0.01), and number of household (*p* = 0.03) were variables associated with MRSA colonization. Age (*p* = 0.05) and receiving government financial assistance (*p* = 0.05) were also included in the multivariable logistic regression analysis as potential confounding factors. In the bivariate regression analysis for all patients, those attending the public clinic and living in a household with an unstable income had significantly higher odds of MRSA colonization (OR = 1.98, 95% CI 1.08–3.65 and OR = 2.45, 95% CI 1.26–4.76, respectively) (Table 3).

**Table 3:** Risk factors for MRSA colonization for all pediatric patients and only for pediatric patients examined at the public clinic

Risk factor for MRSA colonization	All patients				Public clinic only			
	Unadjusted OR	95% CI	Adjusted OR	95% CI	Unadjusted OR	95% CI	Adjusted OR	95% CI
Attends public clinic	1.98	1.08–3.65	2.23	0.94–5.30	NA	NA	NA	NA
Does not have stable income	2.45	1.26–4.76	2.05	0.93–4.55	2.20	1.02–4.77	2.62	0.99–6.98
Monthly household income ($USD)								
$0–$970	1.00	–	1.00	–	1.00	–	1.00	–
$971–$1938	1.97	0.96–4.07	3.40	1.45–7.98	4.44	1.84–10.71	4.84	1.90–12.31
$1939 or more	0.61	0.27–1.40	1.36	0.46–4.04	NA	NA	NA	NA
Self-reported slum residence	0.90	0.47–1.72	–	–	0.52	0.25–1.09	0.40	0.17–0.97
Five years of age or older	1.74	0.97–3.13	–	–	1.85	0.86–3.99	–	–
Receives government financial assistance	1.89	0.99–3.61	–	–	1.51	0.72–3.19	–	–
Five or more household members	1.79	0.98–3.27	–	–	1.25	0.59–2.63	–	–

*OR* Odds ratio, *CI* Confidence interval. Hosmer and Lemeshow Goodness-of-Fit Test *p*-value for all patients model = 0.693, for public clinic only = 0.911. Dash (−) indicates not included in the analysis or not applicable. NA refers to categories for which there were no subjects, and therefore, odd ratios could not to be calculated

However, in the multivariate regression model, neither of these factors remained significant after adjusting for income. Only children from households with a monthly income $971–$1938 had a significantly higher adjusted odds of MRSA colonization in comparison to children from households with a monthly income of $0–$970 (AOR = 3.40, 95% CI 1.45–7.98). In the stratified logistic regression analysis by clinic, no risk factor for MRSA colonization was significant among patients examined at the private clinic (data not shown). For patients examined at the public clinic, living in a household with a monthly income of $971–$1938 continued to be a significant risk factor for MRSA colonization in the adjusted model (AOR = 4.84, 95% CI 1.90–12.31). Slum residence was found to be a protective factor among children who visited the public clinic (AOR = 0.40, 95% CI 0.17–0.97).

### Risk factors for BHS colonization

Among all patients, BHS colonization by any serogroup was associated with age ≥ 5 years and school attendance (*p* < 0.05). Among clinical characteristics for all patients, attending the clinic due to symptoms/illness (*p* = 0.01), having an earache (*p* = 0.03), sore throat (*p* < 0.01), upper respiratory tract infection (*p* = 0.04) or rhinitis (*p* = 0.01) were associated with the presence of BHS (data not shown). Being 5 years of age or older and attending school were found to be significant risk factors for BHS colonization in the multivariate regression analysis (AOR = 16.36, 95% CI 3.77–70.98 and AOR = 6.46, 2.52–16.54, respectively) (Table 4).

**Table 4:** Characteristics associated with the presence of beta-hemolytic streptococcal among pediatric patients examined at private and public clinics – Niterói, RJ, Brazil, 2014

Characteristic	Unadjusted OR	95% CI	Adjusted OR	95% CI
Attends public clinic	1.70	0.73–3.94	–	–
5 years of age or older	19.30	4.49–82.91	16.36	3.77–70.98
Attends school	14.61	3.40–62.71	6.46	2.52–16.54
Employment status				
Employed	1.00	–	–	–
Stay-at-home	0.48	0.11–2.09	–	–
Other	2.83	1.00–8.05	–	–
Attending the clinic due to symptoms/illness (vs. routine)	3.10	1.36–7.10	–	–
Earache	5.33	1.43–19.84	–	–
Sore throat	8.97	3.67–21.89	–	–
Upper respiratory tract infection	2.33	1.00–5.40	–	–
Rhinitis	2.86	1.26–6.52	–	–

Dash (−) indicates not included in the analysis or not applicable

*OR* Odds ratio. Hosmer and Lemeshow Goodness-of-Fit Test *p*-value = 0.414

*CI* Confidence interval

## Discussion

We observed differences in *S. aureus* and BHS colonization among pediatric patients from different SES communities living in the same city in Brazil. Middle/low SES patients were found to be more frequently colonized with these bacteria than high-income and slum resident patients. Bacterial isolates from middle/low-income children had the highest diversity in both MRSA genetic characteristics and BHS serogroups, covering four of the five SCC*mec* types and all BHS serogroups. In Brazil, middle/low SES neighborhoods tend to be on the border of slums, sometimes referred to as the “periphery”, between industrial or commercial zones and high SES neighborhoods. Despite the relation of slum areas with low sanitation resources [14], slum residence was not a risk factor for MRSA colonization among children attended in the public clinic.

Differences in *S. aureus*, MRSA, and *S. pyogenes* colonization prevalence between SES communities in Niterói mirror differences in the prevalence of these pathogens between high-income and low- and middle-income countries (LMIC). Here, *S. aureus* colonization prevalence was 32% among high-income SES children. This frequency is similar to that found in healthy or outpatient children in high-income countries, such as USA (35–37%) [24, 25], Canada (24%) [26], Taiwan (29%) [27], China (28%) [28], as well as in some LMIC, such as India (35%) [29], Botswana (52%) [30], Iran (28%) [31], Gambia (25%) [32] and Angola (44%) [33]. However, the colonization frequency (40%) found among middle/low-income SES group and slum residents was higher than found in most of these countries, but similar to those found among public childcare centers in Brazil (47–48%) [22, 34]. A study in Iran also found lower income to be independently associated with *S. aureus* colonization frequency [31].

MRSA colonization among all SES groups (8.1%) in Niterói was higher than frequencies observed in high-income populations (≤ 1%), such as in USA, Canada, northern Europe, and Hong Kong [28]. However, the difference in colonization prevalence between high and low SES groups observed in the present study (6% vs. 14%) reflects differences in prevalence among children from these high-income populations compared with 6–29% in populations from LMICs, such as Iran, Botswana, Angola, India [29–31, 33, 35], and public childcare centers in Brazil [22, 34].

In general, MRSA strains had low frequency of resistance to other antimicrobial agents, except to erythromycin. SCC*mec* types IV and V are frequently found in CA-MRSA [34] and, in the present study, type IV was found in the majority (84%) of the MRSA strains. The prevalence of PVL-positive strains is usually high among CA-MRSA involved in severe diseases, and may reach as much as 90% [10]. In this study, we detected the PVL genes among 20% of the MRSA strains. Since we focused on colonization isolates, the genotypes responsible for colonization may differ from those associated with invasive/severe diseases.

BHS colonization frequency was low (4%), similar to another study conducted in southern Brazil (5.6%) [35]. However, the small sample size limited our ability to investigate differences in colonization and risk factors by SES. None of the isolates was resistant to any antimicrobial agent tested. Of note, none of the children colonized with GAS had a household history of rheumatic fever, a disease commonly associated with GAS in developing countries [1]. Interestingly, groups B, C and G streptococci, which also colonize cattle, dogs, and other mammals [2], were only found among middle/low SES children and slum residents. Most participants’ legal guardians did not report having animals in the house, but the presence of these bacteria could represent exposure to non-domesticated animals or animal products through poor living conditions or contaminated food.

A major limitation of this study is that some children with symptoms may have the disease caused by the bacteria investigated, especially BHS and sore throat. However, colonization is usually the first step of the infectious process and viruses cause most of the upper respiratory tract diseases. If we excluded symptomatic children from the analysis, we would exclude patients with viral infections. Also, the prevalence of children with symptoms did not differ between subpopulations and, therefore, does not affect the main conclusions of this report. In addition, parents may be more likely to take their children in for routine care when the child has an acute illness, resulting in a higher likelihood of bacterial colonization among our study population. However, these factors are likely to occur in both private and public pediatric populations and we would not expect them to decrease the association with SES found in the present study. Another important limitation was the difference in age between the private and public clinics, given older children are more likely to be colonized with BHS [11]. This was further demonstrated in the present study by age > 5 years and attending school being risk factors for BHS colonization. Finally, the classification of pediatric patients into three SES groups may be a possible limitation, since self-reported residence location and type of clinic attended do not assure the socioeconomic status of the patients; however, the criteria we used to divide the patients into three subpopulations are very well described and support our analyses.

## Conclusions

Our study demonstrates that subpopulations from different SES strata within the same city may have different exposures or risk factors for bacterial transmission and drug resistance. Children living in middle/low SES communities were more likely to be colonized with *S. aureus*, MRSA, and BHS. We also observed variation among BHS serogroups and MRSA SCC*mec* types. While living conditions in slum communities would suggest they are at higher risk, residents who live on the periphery of slums or living at the intersection of extreme poverty and high income were shown to be at higher risk for both bacterial colonization and drug resistance. This study also supports the importance of including a representative sample of different SES subpopulations for research on disease transmission or prevalence studies in cities with high income inequality. Public health officials also should consider differences in transmission across SES subpopulations when developing public health interventions and conducting outbreak investigations for bacterial infections.

## Abbreviations

AGSN: *Aglomerados subnormais*
AOR: Adjusted odds ratio
BHS: Beta-hemolytic streptococci
BRL: Brazilian Real
GAS: Group A streptococci
GBS: Group B streptococci
GDP: Gross domestic product
LMIC: Low- and middle-income countries
MRSA: Methicillin-resistant *Staphylococcus aureus*
OR: Odds ratio
SCC*mec*: Staphylococcal chromosome cassette *mec*
SES: Socioeconomic status
USD: US Dollar
